# Comparison of cardiovascular and renal outcomes between dapagliflozin and empagliflozin in patients with type 2 diabetes without prior cardiovascular or renal disease

**DOI:** 10.1371/journal.pone.0269414

**Published:** 2022-10-17

**Authors:** Jayoung Lim, In-Chang Hwang, Hong-Mi Choi, Yeonyee E. Yoon, Goo-Yeong Cho

**Affiliations:** 1 Department of Cardiology, Cardiovascular Center, Seoul National University Bundang Hospital, Seongnam, Gyeonggi, South Korea; 2 Department of Internal Medicine, Seoul National University College of Medicine, Seoul, South Korea; University of Campania Luigi Vanvitelli: Universita degli Studi della Campania Luigi Vanvitelli, ITALY

## Abstract

**Background:**

Cardiovascular and renal benefits of sodium glucose co-transporter 2 inhibitors (SGLT2i) have been clearly demonstrated. However, studies comparing the effects of dapagliflozin and empagliflozin are scarce. In addition, relatively few studies have analyzed the effects of SGLT2i in diabetic patients without established atherosclerotic cardiovascular disease (ASCVD), chronic kidney disease (CKD), or heart failure (HF), and current guidelines recommend SGLT2i and other antidiabetic drugs equally in this population. Therefore, we aimed to compare the clinical outcomes between dapagliflozin, empagliflozin, and dipeptidyl peptidase-4 inhibitors (DPP4i) in patients with type 2 diabetes without prior ASCVD, CKD, or HF.

**Methods:**

Using a propensity-score matching method, we retrospectively analyzed 921 patients treated with dapagliflozin, 921 patients treated with empagliflozin, and 1842 patients treated with DPP4i (control group). Study outcomes comprised composite coronary events (acute coronary syndrome and coronary revascularization), composite ischemic events (coronary events and stroke), and composite heart failure and renal events.

**Results:**

During follow up (median, 43.4 months), the incidence of composite coronary events was significantly lower in the SGLT2i groups than in the control group, and the incidence of composite ischemic events was lower in the dapagliflozin group than in the control group. Dapagliflozin and empagliflozin both demonstrated significant benefits in terms of HF and renal outcomes, supported by renoprotective effects, as assessed by the change in glomerular filtration rate. At 24–36 months of treatment, the empagliflozin group had higher low-density lipoprotein cholesterol levels, and lower glycated hemoglobin levels, compared to those in the dapagliflozin and control groups.

**Conclusion:**

SGLT2i use was associated with a significantly reduced risk of ASCVD, HF hospitalization, and renal events, compared to that with DPP4i use among diabetic patients without prior ASCVD, CKD, or HF. There were no significant differences in clinical outcomes between dapagliflozin and empagliflozin, supporting a SGLT2i class effect.

## Introduction

Type 2 diabetes is a major risk factor for macrovascular and microvascular diseases [1]. The treatment strategy for type 2 diabetes has evolved from glycemic control to patient-centered approaches, with consideration of the risk of atherosclerotic cardiovascular disease (ASCVD), chronic kidney disease (CKD), and heart failure (HF) [2]. This expansion in therapeutic strategy is mainly based on the introduction of new anti-hyperglycemic agents, such as sodium glucose co-transporter 2 inhibitors (SGLT2i), which were the first to demonstrate a prognostic benefit. Large-scale clinical trials of two SGLT2i, empagliflozin and dapagliflozin, have shown direct and indirect evidence for their significant cardiovascular and renal protective effects in patients with type 2 diabetes [3–9]. Mechanisms of SGLT2i for the clinical benefits include natriuresis and osmotic diuresis, modulation of cardiac metabolism, and restoration of tubuloglomerular feedback [10, 11]. Further, as microvascular dysfunction, inflammation, oxidative stress, and fibrosis lead to the progression of diabetic kidney disease and HF, SGLT2i-mediated attenuations in these pathways may contribute to cardiovascular and renal protection [12–14].

Several meta-analyses have suggested that there is a class effect for SGLT2i [15–18]. However, a few retrospective studies have suggested differences in effects between SGLT2i, favoring the use of dapagliflozin over empagliflozin in terms of HF prevention [19, 20]. On the other hand, a few studies have indicated more favorable glycemic control and management of cardiometabolic parameters with empagliflozin than with dapagliflozin [21, 22]. Despite potential differences in benefit profiles, no trial has directly compared these SGLT2i in terms of clinical outcomes. In addition, relatively few dedicated studies have analyzed the effects of SGLT2i in diabetic patients with low to intermediate ASCVD risk or without established ASCVD, CKD, or HF. Thus, in this population, current guidelines recommend SGLT2i and other antidiabetic drugs equally, with consideration of the hypoglycemia risk and effects on weight loss, not cardiovascular and renal benefits [23]. Given that dipeptidyl peptidase-4 inhibitors (DPP4i) are the most widely used antidiabetic medication following metformin in routine clinical practice [24–26], it would be relevant to compare the effects of SGLT2i with DPP4i in diabetic patients without prior ASCVD, CKD, or HF, in order to assess a class effect for SGLT2i.

Therefore, in the present retrospective study, we aimed to compare the cardiovascular and renal outcomes between dapagliflozin and empagliflozin in patients with type 2 diabetes without prior ASCVD, CKD, or HF, and assess their impact on lipid profiles, glycemic control, and renal function. Additionally, the effects of dapagliflozin and empagliflozin were further compared to those of DPP4i.

## Materials and methods

### Study population

Patients with type 2 diabetes prescribed empagliflozin, dapagliflozin, or a dipeptidyl peptidase-4 inhibitor (DPP4i) at Seoul National University Bundang Hospital from April 2009 to December 2020 were retrospectively identified [27]. The date of medication (SGLT2i or DPP4i) initiation was defined as the index date. Exclusion criteria were type 1 diabetes mellitus, prior HF, prior ASCVD (angina, myocardial infarction, coronary revascularization, peripheral artery disease, and stroke), prior CKD (glomerular filtration rate [GFR] <45 mL/min/1.73m^2^), short duration of medication use (<3 months), low medication possession ratio (<75%), and simultaneous or sequential use of empagliflozin and dapagliflozin.

**Fig 1:**
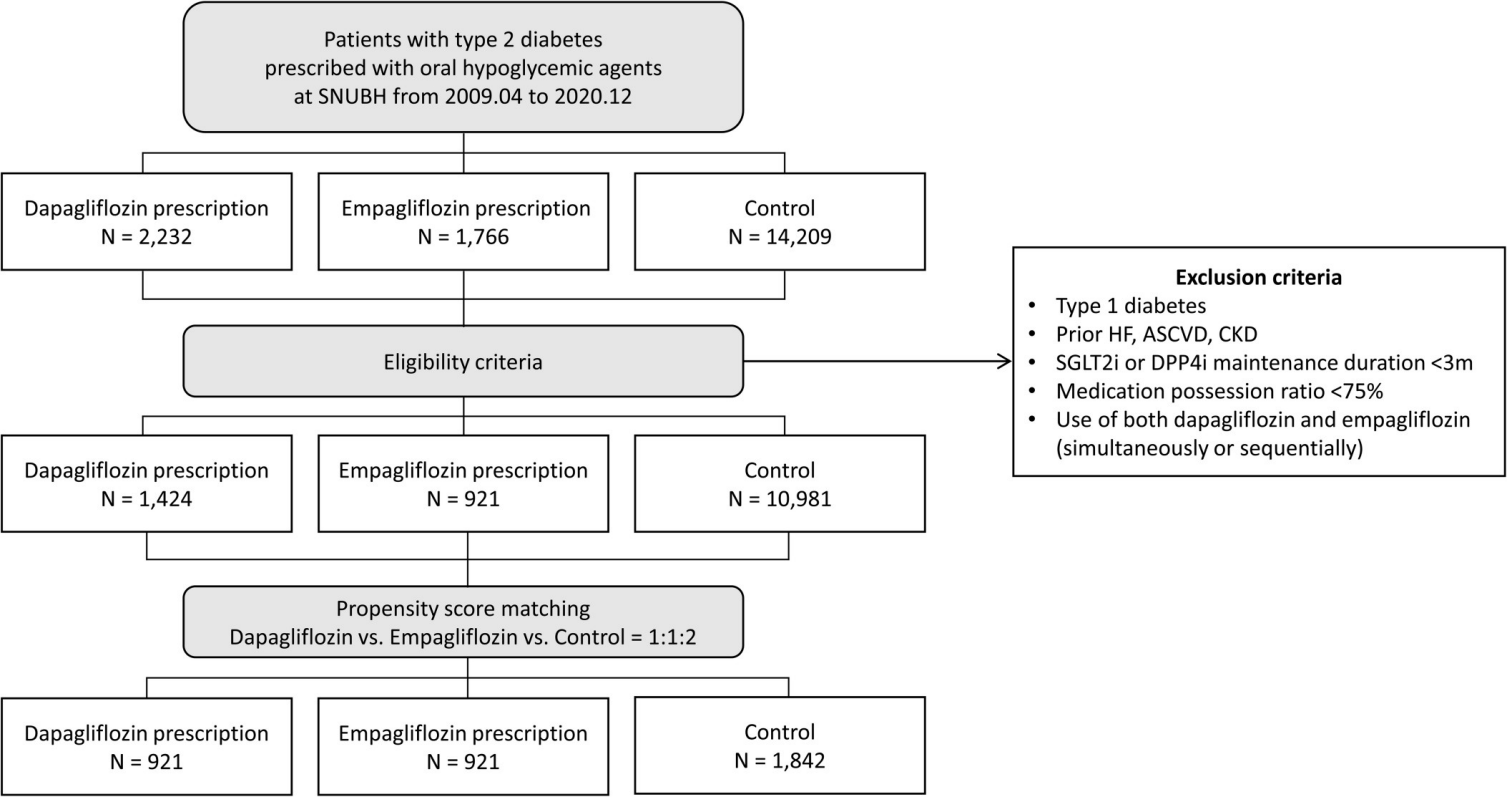
Study flowchart. After 1:1:2 propensity score matching, there were 921 patients treated with empagliflozin, 921 patients treated with dapagliflozin, and 1,842 patients treated with DPP4i (control group). ASCVD, atherosclerotic cardiovascular disease; CKD, chronic kidney disease; DPP4i, dipeptidyl peptidase-4 inhibitor; HF, heart failure; SGLT2i, sodium-glucose co-transporter 2 inhibitor.

In total, 921 patients treated with empagliflozin, 1,424 patients treated with dapagliflozin, and 10,981 patients treated with DPP4i (control group) were identified (**Fig 1**). After propensity-score matching (1:1:2 ratio) on clinical factors, laboratory findings, and medication use, the empagliflozin and dapagliflozin groups comprised 921 patients each, and the DPP4i (control) group comprised 1,842 patients (**Fig 1** and **S1 Appendix**). The Institutional Review Board of Seoul National Bundang Hospital approved the study protocol and waived the requirement for informed consent.

### Measurements

We collected data on age, sex, systolic blood pressure, diastolic blood pressure, history of smoking, preexisting comorbidities, 10-year ASCVD risk, medications, and laboratory findings, including the lipid profile, fasting glucose level, glycated hemoglobin A1c (HbA1c) level, and GFR. The 10-year ASCVD risk among patients without pre-existing cardiovascular disease was calculated by using the Pooled Cohort Equations, with consideration of age, sex, total cholesterol, low-density lipoprotein cholesterol (LDLc), systolic blood pressure, treatment of high blood pressure, diabetes, and smoking status, according to the American College of Cardiology/American Heart Association guidelines [28, 29].

### Outcomes

Study outcomes comprised composite coronary events (acute coronary syndrome and coronary revascularization), composite ischemic events (acute coronary syndrome, coronary revascularization, and stroke), hospitalization for HF (HHF), renal events (renal death, initiation of renal replacement therapy, and admission due to acute kidney injury or CKD progression), and composite HHF and renal events. Changes in the levels of total cholesterol, LDLc, and HbA1c, and GFR during follow up were also assessed.

### Statistical analysis

To adjust for imbalances in the baseline characteristics of patients among empagliflozin, dapagliflozin, and control groups, propensity-score matching, with a 1:1:2 ratio, was performed using the nearest neighbor method, with the following covariates: age, sex, 10-year ASCVD risk, systolic blood pressure, presence of microalbuminuria/proteinuria, HbA1c level, GFR, and the use of antiplatelet agents, renin-angiotensin system blockers, and statins. The distributions of propensity scores and standardized mean differences were calculated to assess the strength of matching.

Categorical variables are presented as numbers with percentages, and continuous variables as medians with interquartile ranges (IQRs). Group comparisons were performed using the χ2 test for categorical variables and analysis of variance for continuous variables. Hazard ratios (HRs) with 95% confidence intervals (CIs) were estimated using the Cox proportional-hazards method. Statistical analyses were performed using SPSS version 22.0 (IBM Co., Armonk, NY, USA) and R version 4.0.2 (The R Foundation for Statistical Computing, Vienna, Austria). Two-sided p-values <0.05 were considered statistically significant.

## Results

### Baseline characteristics

The baseline characteristics, laboratory findings, and use of medications were well balanced among the groups (**Table 1**). The median age was 56, 56, and 57 years in the dapagliflozin, empagliflozin, and control groups, respectively, and two-thirds of the patients were male. The prevalence of current smoking, hypertension, and dyslipidemia was 20%, 60%, and 80%, respectively. The mean 10-year ASCVD risk ranged 10%–11%, without significant inter-group differences. In all three groups, the median HbA1c level was approximately 8.0% and median GFR was approximately 97 mL/min/1.73m^2^, and the prevalence of microalbuminuria or proteinuria was more than 30%. Two-thirds of the patients were on statins, and less than 50% were on renin-angiotensin system blockers. Regarding anti-diabetic medication, more than 97% of the patients were on metformin, more than 40% were on sulfonylurea, and 18% were on insulin therapy.

**Table 1:** Baseline characteristics.

	Dapagliflozin (n = 921)	Empagliflozin (n = 921)	Control (n = 1,842)	p-value
***Clinical characteristics***				
Age (years)	56 (48–65)	56 (48–64)	57 (48–66)	0.321
Male sex	610 (66.2%)	611 (66.3%)	1177 (63.9%)	0.314
Systolic blood pressure (mmHg)	134 (123–149)	134 (122–147)	133 (122–146)	0.113
Diastolic blood pressure (mmHg)	79 (70–88)	79 (71–88)	78 (71–87)	0.406
Body-mass index (kg/m^2^)	26.0 (24.0–28.7)	26.3 (24.0–28.9)	25.1 (22.7–27.7)	0.061
Current smoker	167 (18.1%)	186 (20.2%)	385 (20.9%)	0.228
Hypertension	541 (58.7%)	538 (58.4%)	1131 (61.4%)	0.215
Dyslipidemia	734 (79.7%)	702 (76.2%)	1462 (79.4%)	0.110
Atrial fibrillation	30 (3.3%)	28 (3.0%)	70 (3.8%)	0.541
10-year ASCVD risk (%)	10.6 (4.2–22.6)	10.4 (4.5–22.6)	11.4 (4.5–24.2)	0.513
***Laboratory findings***				
Total cholesterol (mg/dL)	159 (138–190)	161 (140–190)	161 (134–197)	0.642
HDL cholesterol (mg/dL)	47 (40–54)	46 (40–54)	46 (39–53)	0.057
LDL cholesterol (mg/dL)	104 (85–126)	106 (86–128)	102 (79–131)	0.125
Fasting glucose (mg/dL)	157 (128–189)	158 (128–199)	159 (127–208)	0.110
Hemoglobin A1c (%)	8.1 (7.3–9.1)	8.3 (7.3–9.5)	8.0 (7.1–9.5)	0.087
Creatinine (mg/dL)	0.8 (0.7–0.9)	0.8 (0.7–0.9)	0.8 (0.6–0.9)	0.745
Glomerular filtration rate (mL/min/1.73 m^2^)	96.6 (85.3–106.0)	96.7 (84.9–105.9)	96.5 (83.7–106.4)	0.700
Microalbuminuria or proteinuria	335 (36.4%)	336 (36.5%)	640 (34.7%)	0.565
***Medications***				
Antiplatelet agents	231 (25.1%)	220 (23.9%)	471 (25.6%)	0.629
Statins	615 (66.8%)	600 (65.1%)	1196 (64.9%)	0.615
Calcium channel blockers	258 (28.0%)	246 (26.7%)	558 (30.3%)	0.120
RAS blocker	449 (48.8%)	423 (45.9%)	835 (45.3%)	0.226
Beta blockers	88 (9.6%)	77 (8.4%)	153 (8.3%)	0.515
Diuretics	107 (11.6%)	122 (13.2%)	247 (13.4%)	0.393
Direct oral anticoagulants	22 (2.4%)	20 (2.2%)	55 (3.0%)	0.392
Insulin	168 (18.2%)	166 (18.0%)	341 (18.5%)	0.950
Metformin	897 (97.4%)	896 (97.3%)	1789 (97.1%)	0.913
Sulfonylurea	407 (44.2%)	392 (42.6%)	763 (41.4%)	0.379
Thiazolidinedione	81 (8.8%)	80 (8.7%)	144 (7.8%)	0.594
GLP1 receptor agonists	2 (0.2%)	2 (0.2%)	8 (0.4%)	0.596

Values are medians with interquartile ranges (IQR; Q1–Q3).

ASCVD, atherosclerotic cardiovascular disease; GLP1, glucagon-like peptide 1; HDL, high-density lipoprotein; HHF, hospitalization for heart failure; LDL, low-density lipoprotein; RAS, renin-angiotensin system

### Clinical outcomes

**Fig 2:**
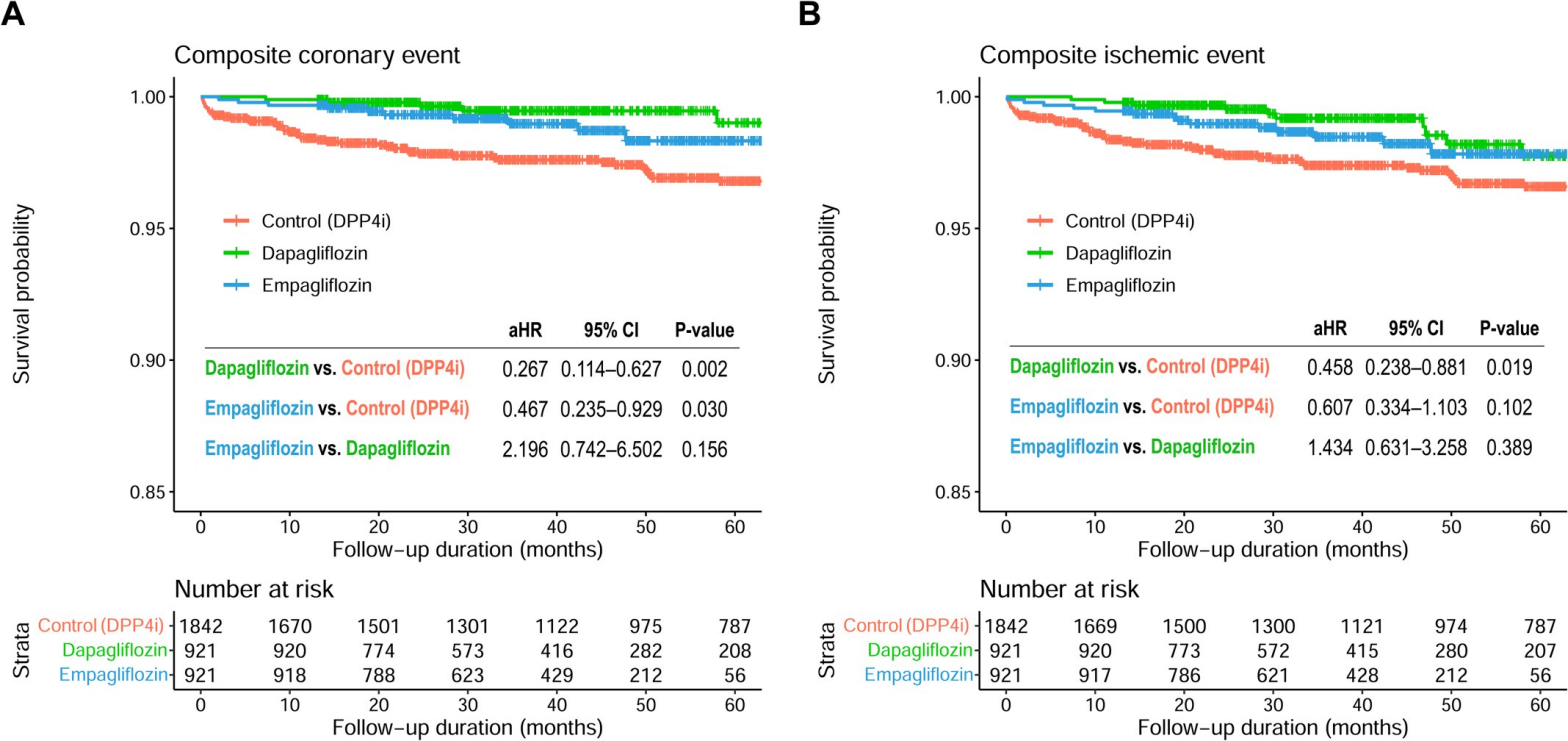
Event-free survival curves for atherosclerotic events. Kaplan-Meier curves comparing the risk of **(A)** composite coronary events and **(B)** composite ischemic events between the 3 groups. CI, confidence interval; HR, hazard ratio; SGLT2i, sodium-glucose co-transporter 2 inhibitor.

The median follow-up duration was 37.3 months (IQR, 24.5–55.7 months), 37.1 months (IQR 27.1–49.2 months), and 55.1 months (27.5–84.9 months) in the dapagliflozin, empagliflozin, and control groups, respectively (**Table 2**). Composite coronary events occurred in 6 patients (0.7%) in the dapagliflozin group, 10 patients (1.1%) in the empagliflozin group, and 55 patients (3.0%) in the control group. On multivariable analysis, advanced age, current smoking, presence of hypertension, and higher 10-year ASCVD risk were associated with a higher risk of composite coronary and ischemic events (**Table 3**). SGLT2i use was significantly associated with a lower incidence of composite coronary events (dapagliflozin vs. control: HR, 0.267; 95% CI, 0.114–0.627; p = 0.002) (empagliflozin vs. control: HR, 0.467; 95% CI, 0.235–0.929; p = 0.030), without a significant difference between dapagliflozin and empagliflozin groups (HR, 2.196; 95% CI, 0.742–6.502; p = 0.156) (**Fig 2A**). Likewise, compared to that in the control group, the occurrence of composite ischemic events was significantly less frequent in the dapagliflozin group, and tended to be lower in the empagliflozin group, without a significant difference between dapagliflozin and empagliflozin groups (**Fig 2B**).

**Table 2:** Clinical outcomes.

	Dapagliflozin (n = 921)	Empagliflozin (n = 921)	Control (n = 1,842)	p-value
Follow-up duration (months)	37.3 (24.5–55.7)	37.1 (27.1–49.2)	55.1 (27.5–84.9)	<0.001
All-cause death	2 (0.2%)	2 (0.2%)	60 (3.3%)	<0.001
Cardiovascular death	1 (0.1%)	2 (0.2%)	5 (0.3%)	0.897
Acute coronary syndrome	2 (0.2%)	2 (0.2%)	31 (1.7%)	<0.001
Coronary revascularization	5 (0.5%)	7 (0.8%)	39 (2.1%)	0.001
Stroke	3 (0.3%)	2 (0.2%)	4 (0.2%)	0.908
HHF	3 (0.3%)	6 (0.7%)	40 (2.2%)	<0.001
Renal events (renal admission, progression to ESRD)	2 (0.2%)	2 (0.2%)	30 (1.6%)	<0.001
Composite coronary events	6 (0.7%)	10 (1.1%)	55 (3.0%)	<0.001
Composite ischemic events	11 (1.2%)	14 (1.5%)	59 (3.2%)	0.001
Composite of HHF and renal events	4 (0.4%)	8 (0.9%)	64 (3.5%)	<0.001

ESRD, end-stage renal disease; HHF, hospitalization for heart failure

**Table 3:** Multivariable predictors of ischemic events.

	Composite coronary events*			Composite ischemic events†		
	Adjusted HR	95% CI	p-value	Adjusted HR	95% CI	p-value
Age (per +1 year)	1.024	1.004–1.045	0.021	1.026	1.007–1.045	0.006
Current smoker	1.788	1.077–2.968	0.025	2.039	1.295–3.213	0.002
Hypertension	3.611	1.831–7.122	<0.001	3.081	1.690–5.615	<0.001
10-year ASCVD risk (per +1%)	1.024	1.012–1.035	<0.001	1.014	1.003–1.026	0.015
Antidiabetic medication						
• Control (DPP4i)	*Reference*			*Reference*		
• Dapagliflozin	0.267	0.114–0.627	0.002	0.458	0.238–0.881	0.019
• Empagliflozin	0.467	0.235–0.929	0.030	0.607	0.334–1.103	0.102

Univariable factors with p-values <0.200 were entered into the multivariable Cox proportional hazard regression analysis using the stepwise backward elimination method.

* Composite coronary events included acute coronary syndrome and coronary revascularization.

† Composite ischemic events included acute coronary syndrome, coronary revascularization, and stroke.

ASCVD, atherosclerotic cardiovascular disease; CI, confidence interval; DPP4i, dipeptidyl peptidase-4 inhibitor; HR, hazard ratio

**Fig 3:**
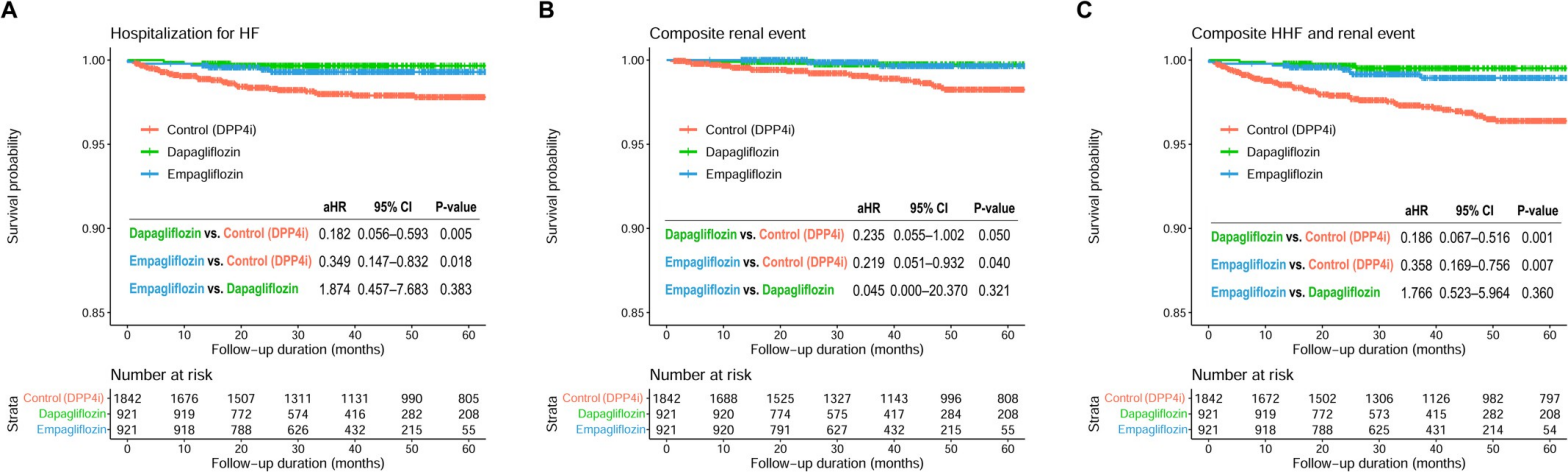
Event-free survival curves for HHF and renal events. Kaplan-Meier curves comparing the risk of **(A)** HHF, **(B)** composite renal events, and **(C)** composite HHF and renal events between the 3 groups. CI, confidence interval; HHF, hospitalization for heart failure; HR, hazard ratio; SGLT2i, sodium-glucose co-transporter 2 inhibitor.

Composite HHF and renal events occurred in 4 patients (0.4%) in the dapagliflozin group and 8 patients (0.9%) in the empagliflozin group (**Table 2**). Significant risk factors for HHF and renal events comprised atrial fibrillation, impaired renal function, and the presence of microalbuminuria or proteinuria (**Table 4**). Both dapagliflozin and empagliflozin demonstrated significant beneficial effects against HHF and renal events compared to that with DPP4i (dapagliflozin vs. control: HR, 0.186; 95% CI, 0.067–0.516; p = 0.001) (empagliflozin vs. control: HR, 0.358; 95% CI, 0.169–0.756; p = 0.007) (**Fig 3**).

**Table 4:** Multivariable predictors of HF and renal events.

	HHF			Renal events*			Composite of HHF and renal events		
	Adjusted HR	95% CI	p-value	Adjusted HR	95% CI	p-value	Adjusted HR	95% CI	p-value
Age (per +1 year)	1.022	0.998–1.048	0.076	-	-	-	-	-	-
Hypertension	1.961	0.960–4.003	0.064	-	-	-	-	-	-
Atrial fibrillation	6.888	3.398–13.961	<0.001	-	-	-	4.431	2.355–8.337	<0.001
HbA1c (per +1%)	1.145	1.002–1.309	0.047	0.786	0.622–0.994	0.044	-	-	-
GFR (per +1 mL/min/1.73m^2^)	-	-	-	0.971	0.954–0.988	0.001	0.983	0.972–0.995	0.005
Microalbuminuria or proteinuria	1.668	0.928–2.996	0.087	2.993	1.444–6.205	0.003	2.225	1.402–3.531	0.001
Antidiabetic medication									
• Control (DPP4i)	*Reference*			*Reference*			*Reference*		
• Dapagliflozin	0.182	0.056–0.593	0.005	0.235	0.055–1.002	0.050	0.186	0.067–0.516	0.001
• Empagliflozin	0.349	0.147–0.832	0.018	0.219	0.051–0.932	0.040	0.358	0.169–0.756	0.007

Univariable factors with p-values <0.200 were entered into the multivariable Cox proportional hazard regression analysis using the stepwise backward elimination method.

* Renal events included renal death, hospitalization for acute kidney injury, and progression to end-stage renal disease.

CI, confidence interval; DPP4i, dipeptidyl peptidase-4 inhibitor; GFR, glomerular filtration rate; HbA1c, glycated hemoglobin A1c; HHF, hospitalization for heart failure; HR, hazard ratio

### Laboratory findings

**Fig 4:**
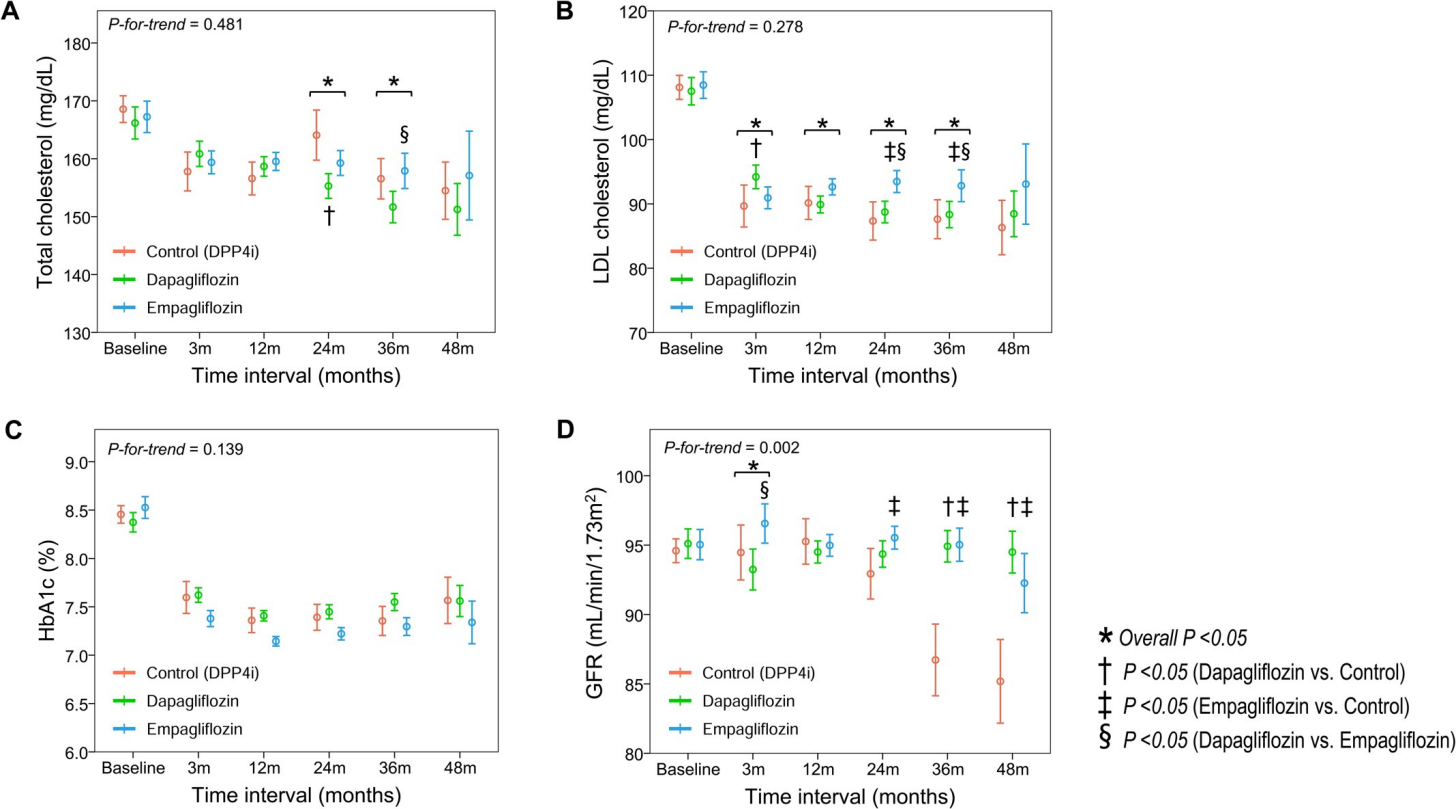
Changes in laboratory findings. Serial changes in **(A)** total cholesterol, **(B)** LDL cholesterol, **(C)** HbA1c, and **(D)** GFR are plotted according to group. GFR, glomerular filtration rate; HbA1c, glycosylated hemoglobin; LDL, low-density lipoprotein; SGLT2i, sodium-glucose co-transporter 2 inhibitor.

Changes in the levels of total cholesterol, LDLc, and HbA1c, and GFR during follow up are shown according to group in **Fig 4**. At baseline, there were no significant inter-group differences in the levels of total cholesterol, LDLc, and HbA1c, and GFR. Overall, the total cholesterol level did not show inter-group differences during follow up (**Fig 4A**). However, at 24 and 36 months of treatment, the LDLc level was significantly higher in the empagliflozin group than in the control and dapagliflozin groups (**Fig 4B**), and the HbA1c level was significantly lower in the empagliflozin group than in the other groups (**Fig 4C**). Additionally, the GFR level tended to be better preserved at 24 months of treatment, and was significantly higher at 36 months and 48 months of treatment, in the dapagliflozin and empagliflozin groups than in the control group (**Fig 4D**).

## Discussion

In this retrospective study, we compared cardiovascular and renal outcomes among dapagliflozin, empagliflozin, and DPP4i (control) groups in 3,684 patients with type 2 diabetes and without prior ASCVD, HF, and CKD. Patients treated with SGLT2i demonstrated a significantly lower risk of atherosclerotic events, HHF, and renal events than that in patients treated with DPP4i (control group), with significant benefits in renal protection as assessed by the GFR level. There were no significant differences in clinical outcomes between dapagliflozin and empagliflozin groups. Of note, at 24–36 months of treatment, we observed higher LDLc levels and lower HbA1c levels in the empagliflozin group than in the other groups.

### Clinical benefits of SGLT2i

A series of landmark trials provided robust evidence on the cardiovascular and renal benefits of SGLT2i, regardless of baseline ASCVD status. In the EMPA-REG outcome trial, empagliflozin was associated with a reduced risk of cardiovascular death (HR, 0.86; 95% CI, 0.74 to 0.99; p = 0.04 for superiority) in patients with type 2 diabetes and established ASCVD [6]. Canagliflozin showed a reduced risk for HHF and cardiovascular death (HR, 0.78; 95% CI, 0.52–0.87; p = 0.02) and composite renal outcomes (HR, 0.60; 95% CI, 0.47 to 0.77; p<0.001) in patients with type 2 diabetes, two-thirds of whom had established ASCVD [3, 4]. In the DECLARE–TIMI 58 trial, dapagliflozin showed a lower rate of cardiovascular death and HHF compared to that with placebo in patients with type 2 diabetes with established ASCVD (40%) or at high risk for ASCVD (60%) (HR, 0.83; 95% CI, 0.73–0.95; p = 0.005) [5]. In a meta-analysis, SGLT2i use was associated with a reduced risk of major adverse cardiovascular events, HHF, and adverse kidney outcomes, independent of baseline ASCVD status [30].

Furthermore, the benefits of SGLT2i use have been observed in patients without prior cardiovascular disease, HF, or CKD. In the EMPRISE East Asia study, empagliflozin treatment was associated with a 28% reduced risk for HHF in diabetic patients without cardiovascular disease [31]; SGLT2i use showed a 36% risk reduction for HHF among diabetic patients without prior HF [32]; and SGLT2i use resulted in a 30%–40% reduction in the risk of HHF and 40%–50% reduction in the risk of adverse renal outcomes among diabetic patients with GFR ≧60 mL/min/1.73m^2^ [33]. These findings support the benefits of SGLT2i for primary prevention in patients with type 2 diabetes but without overt ASCVD, HF, or CKD.

The benefits of SGLT2i observed in clinical trials can be explained by multiple mechanisms, beyond the glucose lowering effect: improvement in ventricular loading condition by natriuresis and osmotic diuresis, improvement in cardiac metabolism, reduced myocardial necrosis and fibrosis, and restoration of tubuloglomerular feedback [10, 11]. In addition, the pleiotropic effects of SGLT2i include benefits on endothelial function by attenuating oxidative stress and inflammation, and reductions in plaque size and vulnerability, as shown in preclinical and clinical studies [14, 34].

### Direct comparisons between dapagliflozin and empagliflozin

Given the concordant benefits of various SGLT2i, as well as their similar pharmacologic profiles, a class effect has been suggested for this drug entity. Although there have been some discrepancies in the reported clinical outcomes in clinical trials, this can be explained by differences in the inclusion criteria, baseline characteristics, and outcome definitions. However, several studies have suggested that SGLT2i effects may differ according to drug type. According to a multi-institutional cohort study by Shao et al., dapagliflozin may offer a more favorable benefit in terms of HF prevention, compared to that with empagliflozin [19]. In another study by Shao et al., dapagliflozin had a more favorable HHF risk reduction than empagliflozin in patients with type 2 diabetes without ASCVD, while similar HHF risks were observed in patients with type 2 diabetes with ASCVD [20]. The more potent effects of dapagliflozin over empagliflozin for HF outcomes can be explained by the SGLT2:SGLT1 receptor selectivity ratio, which is lower for dapagliflozin (1200:1) than for empagliflozin (2500:1) [35, 36]. More specifically, myocardial ischemia and hypertrophy are associated with SGLT1 upregulation in the myocardium, where SGLT2 receptors are never expressed. This finding suggests that the SGLT2i with a lower specificity for SGLT2 receptors, and thus, a greater effect on SGLT1, has an even greater beneficial effect on HF prevention [37]. In addition, compared to that with empagliflozin, dapagliflozin did not increase plasma aldosterone and noradrenaline levels, which could be advantageous for HF prevention [38].

In the present study, the benefits for HF and renal outcomes did not differ between dapagliflozin and empagliflozin groups, showing consistently lower event rates compared to the DPP4i group. The changes in GFR also demonstrated similar patterns for these two SGLT2i, which showed significant benefits over DPP4i treatment (control group). These findings infer that the use of SGLT2i, regardless of subtype, provides consistent benefits on HF and renal outcomes, suggesting the presence of class effect of SGLT2i. Of note, in contrast to the studies by Shao et al. [19, 20], we did not observe a more favorable benefit with dapagliflozin than with empagliflozin in terms of HF and renal outcomes. However, we acknowledge that the present study had a relatively small sample size and was performed in a retrospective manner. Although our findings support a SGLT2i class effect on HF and renal outcomes, future studies are warranted to compare their effects in a prospective trial or using nationwide real-world data.

Regarding the risk of ischemic events, a prospective observational study by Ku et al. found that empagliflozin resulted in better glycemic control and improvements in cardiometabolic components than that with dapagliflozin among patients with type 2 diabetes [21, 22]. Similarly, in the present study, we found that HbA1c levels tended to be lower in the empagliflozin group than in the dapagliflozin and control groups. However, better glycemic control with empagliflozin was not associated with clinical benefits: the risk of composite coronary and ischemic events was not significantly different between the two SGLT2i groups. Rather, the LDLc levels at 24 and 36 months were significantly higher in the empagliflozin group than in the other groups. This might be a reason for the slightly higher rates of ischemic events in the empagliflozin group than in the dapagliflozin group. Considering that a high LDLc level is a well-established risk factor for cardiovascular events, the possible association between a worse lipid profile and numerically higher rates of ischemic events in the empagliflozin group should be clarified in future clinical trials.

Nonetheless, given the similar clinical outcomes between dapagliflozin and empagliflozin in the present study, as well as the concordantly better outcomes with both SGLT2i compared to that with DPP4i, the present findings support a SGLT2i class effect. Indeed, current guidelines recommend both SGLT2i and DPP4i, equally, as second-line antidiabetic medication in diabetic patients without high ASCVD risk or established ASCVD, CKD, or HF [2]. Considering the characteristics of the present cohort, which comprised diabetic patients with intermediate ASCVD risk, but without established ASCVD, CKD, or HF, the present findings suggest that the use of SGLT2i is preferred even in this population. Further, our results support the pleiotropic effects of SGLT2i for ASCVD prevention, that are independent of the management of glucose and lipid profile.

### Study limitations

First, due to the relatively small sample size with a short follow-up duration, the number of clinical events might not have been adequate to demonstrate a difference between dapagliflozin and empagliflozin. This may also be related to the characteristics of the study population, in which patients with prior ASCVD, CKD, or HF were excluded. Thus, the interpretation of the present findings requires caution, and further studies are needed to assess the comparative effects of dapagliflozin, empagliflozin, and other SGLT2i in higher-risk populations over a longer study period. Second, laboratory tests were not performed with pre-specified schedules due to the retrospective nature of the study. However, more than 16,000 laboratory test results were included in the analysis, suggesting that our findings reflect real-world clinical practice. Third, although we used propensity-score matching to minimize differences in baseline characteristics between groups, some unmeasured confounders might remain unresolved.

## Conclusions

SGLT2i use was associated with a significantly reduced risk of ASCVD, HHF, and renal events compared to that with DPP4i use among diabetic patients without prior ASCVD, HF, or CKD. There were no significant differences between dapagliflozin and empagliflozin, supporting a SGLT2i class effect.

## Supporting information

S1 Appendix Study data. (XLSX)

## Abbreviations

ASCVD: atherosclerotic cardiovascular disease
CI: confidence interval
CKD: chronic kidney disease
DPP4i: dipeptidyl peptidase-4 inhibitor
GFR: glomerular filtration rate
HbA1c: glycated hemoglobin A1c
HF: heart failure
HHF: hospitalization for heart failure
HR: hazard ratio
IQR: interquartile range
LDLc: low-density lipoprotein cholesterol
SGLT2i: sodium glucose co-transporter 2 inhibitors
